# Docetaxel for recurrent adult granulosa cell tumor enabling tertiary cytoreduction

**DOI:** 10.1016/j.gore.2026.102216

**Published:** 2026-09-22

**Authors:** Maryam Ali, Katherine Jane C. Chua, Hui Chen

**Affiliations:** Department of Obstetrics and Gynecology, University of California Davis Medical Center, Sacramento, CA, USA

**Keywords:** Ovarian cancer, Granulosa cell tumor, Taxanes, Case report

## Abstract

**Background:**

Adult granulosa cell tumors (AGCTs) are rare ovarian sex cord-stromal tumors characterized by an indolent clinical course, frequent late recurrences, and limited evidence to guide systemic therapy for recurrent disease. Although platinum- and taxane-based regimens have demonstrated activity, optimal treatment after multiple recurrences remains undefined.

**Case:**

We report a 53-year-old woman with recurrent AGCT who had progressed on multiple chemotherapy and hormonal regimens over eight years. Despite prior exposure to and progression on paclitaxel, her disease responded to single-agent docetaxel for 36 cycles, reducing disease burden sufficiently to enable tertiary cytoreductive surgery. The treatment was well tolerated.

**Conclusion:**

This case highlights the potential activity of docetaxel in recurrent AGCT and supports its consideration as an alternative taxane, particularly for patients who are not candidates for or have progressed after other systemic therapies including paclitaxel. Reports describing the use of docetaxel in AGCT are limited, with only isolated cases published to date. Given the rarity of AGCT, individual case reports remain a valuable source of evidence to inform treatment decisions. Prospective studies are needed to better define the role of docetaxel in recurrent AGCT.

## Highlights

• Single-agent docetaxel may be effective in recurrent AGCT.
• Docetaxel achieved a prolonged response despite prior paclitaxel exposure.
• Durable response to docetaxel enabled tertiary cytoreductive surgery.

## 1 Introduction

Adult granulosa cell tumors (AGCT) are rare ovarian neoplasms arising from sex cord-stromal tissue and make up 2–5% of ovarian malignancies. They are often characterized by their indolent nature and unpredictable recurrence patterns, making long-term management and surveillance challenging (Teoh et al., 2010). For both primary and recurrent AGCT, surgical resection is the preferred treatment. First-line therapy for advanced or recurrent disease includes regimens such as bleomycin/etoposide/cisplatin or carboplatin/paclitaxel (Brown et al., 2024). However, due to the rarity of this disease, limited prospective data, and variable response to conventional chemotherapy regimens, systemic treatment for recurrent disease is less clearly defined.

We report a case of recurrent heavily treated AGCT with prolonged response to single-agent docetaxel, resulting in decreased tumor burden that enabled tertiary cytoreductive surgery. This case highlights that docetaxel is a potential therapeutic option in select patients with recurrent AGCT after multiple prior lines of systemic therapy, including paclitaxel.

## 2 Case presentation

Our patient is a 53-year-old female who initially presented at age 37 (2009) with endometrial intraepithelial neoplasia and a left adnexal mass. She underwent fertility-sparing surgery including a laparoscopic left salpingo-oophorectomy, pelvic lymph node dissection, omental, peritoneal, right fallopian tube biopsies, and a dilation and curettage. Pathology revealed a Stage IA adult granulosa cell tumor of the left ovary. She did not receive any adjuvant treatment and underwent surveillance.

In 2014, the patient experienced her first recurrence with rising inhibin B levels and imaging demonstrating diffuse peritoneal carcinomatosis, including disease in the pelvis, omentum, small bowel mesentery, and liver. She underwent a secondary cytoreductive procedure via an exploratory laparotomy, total abdominal hysterectomy, right salpingo-oophorectomy, diaphragmatic stripping, partial hepatectomy, and argon beam coagulation with 1 cm residual nodal disease densely adherent to the obturator nerve. The patient developed progression of disease on adjuvant chemotherapy with carboplatin and paclitaxel (changed to nab-paclitaxel due to hypersensitivity reaction). She was switched to bleomycin, etoposide and cisplatin (changed to carboplatin due to ototoxicity) for six cycles and had a complete response. Over the course of the next eight years, her disease distribution was not amenable to another cytoreductive surgery and she progressed through multiple lines of anti-hormonal therapy and chemotherapy (Table 1) (Fig. 1).

**Table 1:** Summary of systemic treatment regimens, reason for discontinuation of each regimen, and corresponding inhibin B levels. Systemic Treatment Regimens of our patient with AGCT.

Treatment Regimen	Start of Therapy – End of Therapy (Month/Year)	Reason for Discontinuation	Inhibin B at Start of Therapy	Inhibin B at End of Therapy
Carboplatin and Taxane^1^	12/2014–3/2015	Progressive disease	55	110
Bleomycin, Etoposide and Platinum^2^	3/2015–8/2015	Complete response	110	23
Megestrol Acetate	3/2016–9/2016	Progressive disease	87	170
Anastrozole	9/2016–10/2016	Progressive disease	170	359
Pegylated Liposomal Doxorubicin and Bevacizumab	11/2016–4/2017	Grade 2–3 palmar plantar erythrodysesthesia	359	255
Bevacizumab	6/2017–7/2017	Held due to need for several dental procedures for several months^3^	292	274
Letrozole	8/2017–6/2019	Progressive Disease	327	697
Megestrol Acetate and Tamoxifen	6/2019–9/2019^4^	Progressive disease	697	1093
Fulvestrant	5/2020–8/2020	Progressive disease	5260	5685
Palbociclib and Fulvestrant	8/2020–9/2020	Discontinued for hemorrhage of a mesenteric implant	6250	N/A
Leuprolide Acetate	11/2020–4/2021	Progressive Disease	5985	> 5690^5^
Carboplatin, Etoposide, Leuprolide Acetate	4/2021–5/2021	Progressive Disease	> 5690	> 5690
Cisplatin and Etoposide^6^	5/2021–9/2021	Ototoxicity and neuropathy secondary to cisplatin	> 5690	5360
Etoposide and Leuprolide Acetate	10/2021–12/2021	Progressive disease	> 5610	> 5610
Docetaxel	2/2022–2/2025	Progressive Disease^7^	> 5610	2734
Letrozole	4/2025 - present	N/A	204	273 (*most recent)*

1 Initially started on paclitaxel, switched to nab-paclitaxel due to hypersensitivity reaction.

2 Initially on cisplatin, switched to carboplatin due to ototoxicity.

3 Bevacizumab held for several months of planned dental procedures and letrozole started instead. Given the response to letrozole, bevacizumab was never restarted.

4 7 month treatment break after progression. Hospitalization in 4/2020 with urosepsis and large bowel obstruction revealed further progression.

5 Upper limit of assay.

6 Initially dose reduced for cisplatin before ultimately being discontinued.

7 Demonstrated durable partial response with stable disease. Oligo-progression after 11 cycles with psoas lesion fluctuating in size. In February and March 2025, CT showed multiple sites of progression and decision was made to pursue tertiary debulking before disease burden became unresectable.

**Fig. 1:**
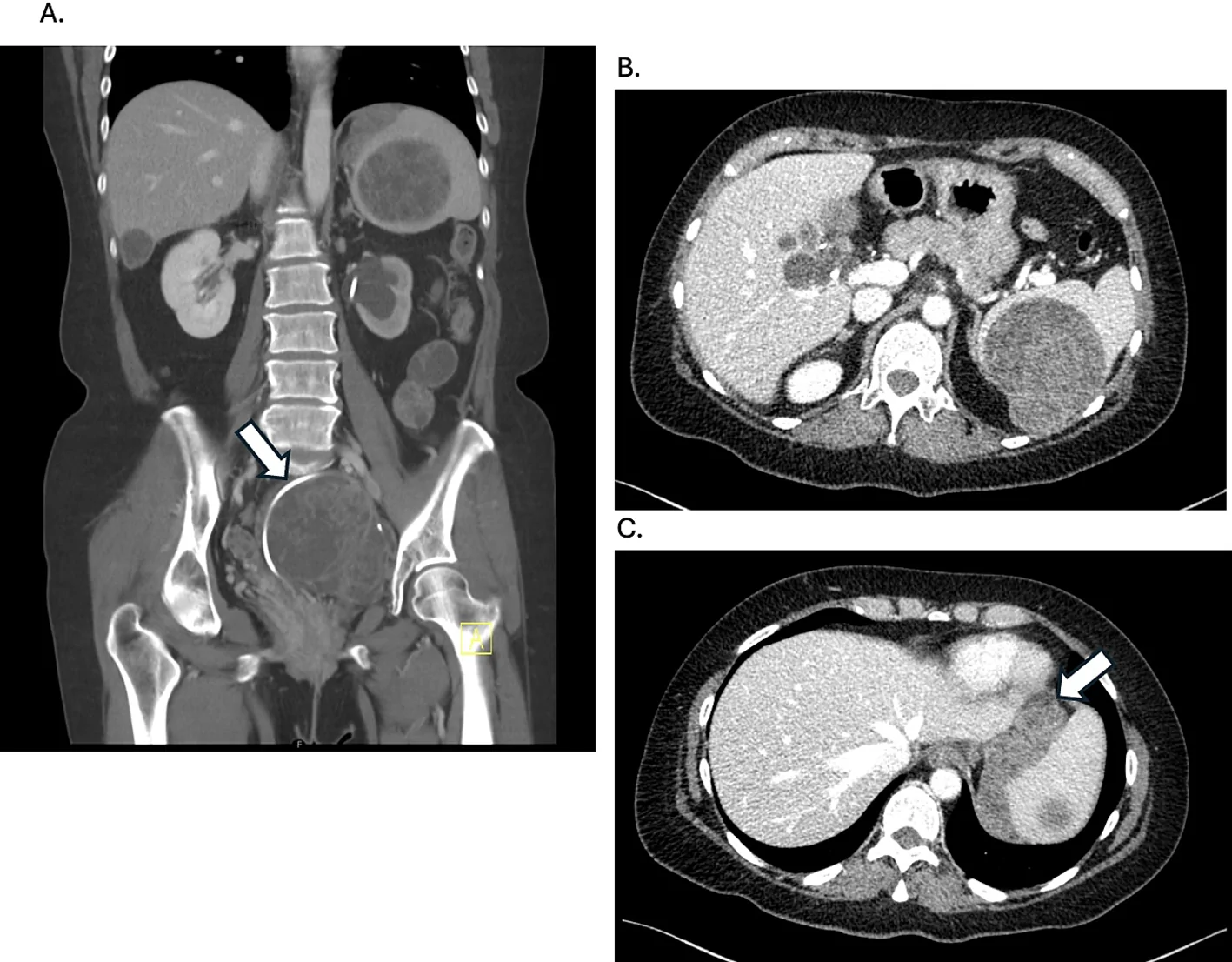
Images from CT abdomen and pelvis with contrast prior to starting docetaxel. (A) Multiple pelvic masses abutting the rectum and encasing bilateral ureters (arrow indicates left ureteral stent), largest measuring 12 × 9 cm; peritoneal carcinomatosis. (B) Multiple hepatic metastases, largest measuring 3.7 × 2.4 cm; dominant subcapsular splenic mass measuring 8.1 × 7.6 cm. (C) Extracapsular medial splenic metastasis measuring 8.6 × 3.7 cm (arrow).

In 2022, the patient was initiated on weekly single-agent docetaxel 30 mg/m^2^ every 28 days, three weeks on with one week off. Docetaxel was selected given its lower rate of neurotoxicity in the setting of her pre-existing chemotherapy-induced peripheral neuropathy. She demonstrated both a radiographic response and a marked decline in inhibin B levels from >5610 pg/mL to a nadir of 550 pg/mL after 11 cycles. She continued docetaxel for a total of 36 cycles with minimal toxicity, maintaining a partial response until eventual disease progression. The partial response reduced the disease burden to an extent which allowed for tertiary cytoreduction (Fig. 2). She underwent an exploratory laparotomy with extensive tumor debulking, splenectomy, left nephrectomy, and radiofrequency ablation of a hepatic lesion with the only site of residual disease being a 2 cm tumor within the left obturator space densely adherent to the left external iliac vessels. Her inhibin B level decreased to 204 pg/mL after surgery.

**Fig. 2:**
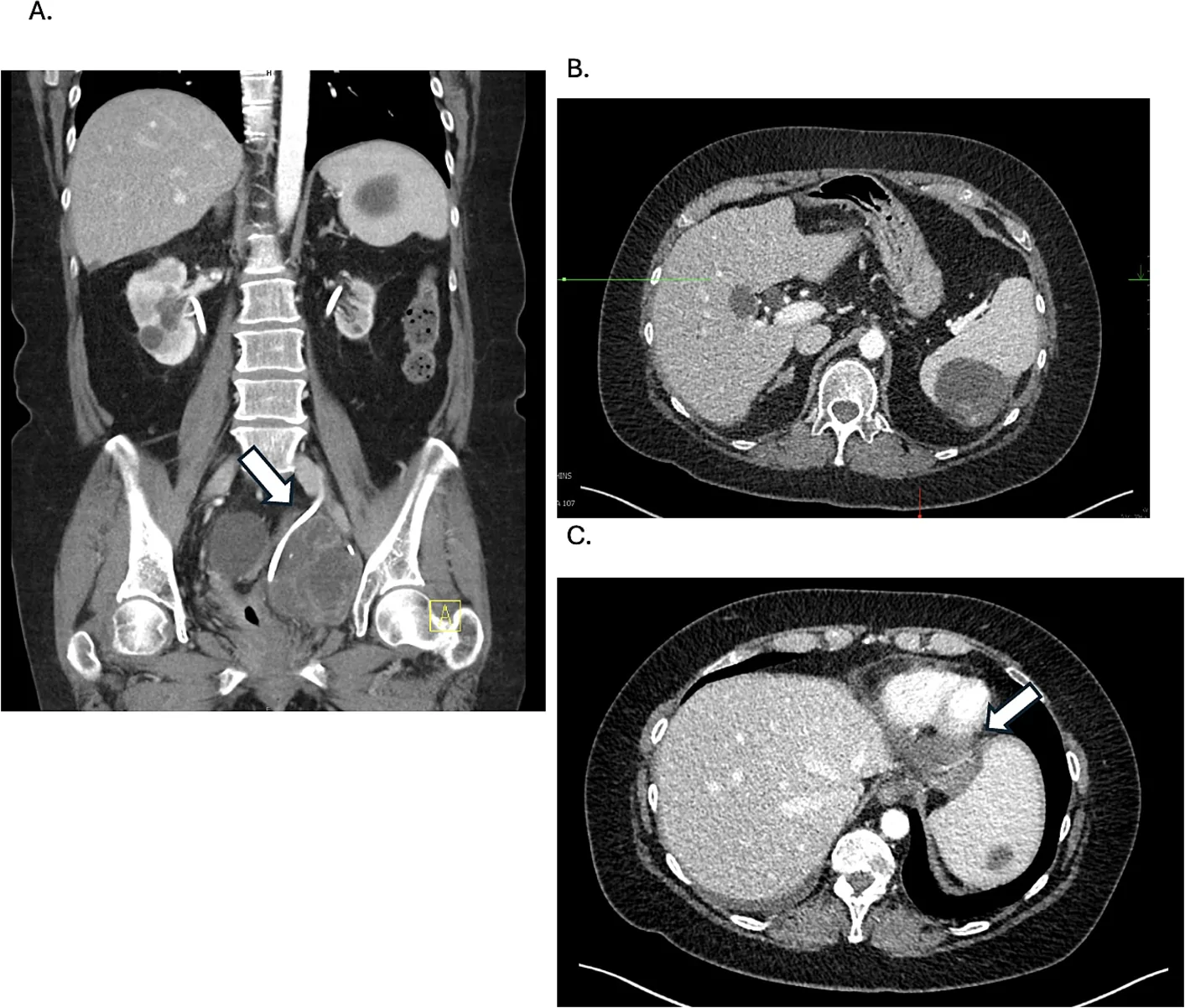
Images from CT abdomen and pelvis with contrast after completing 36 cycles of docetaxel, before tertiary cytoreductive surgery. (A) Multiple pelvic masses, largest measuring 9.6 × 6.9 cm, left ureter coursing along the left pelvic mass (arrow). (B) Hepatic lesions, largest measuring 1.2 cm; dominant subcapsular splenic metastasis measuring 5.7 × 5.2 cm. (C) Extracapsular medial splenic metastasis measuring 4.0 × 1.2 cm.

Imaging obtained two months after surgery demonstrated subtle growth of scattered peritoneal implants and subcapsular hepatic disease, as well as increased subdiaphragmatic nodularity and a right pelvic implant. She was started on letrozole 2.5 mg daily. She is now 13 months from her tertiary cytoreduction, and imaging continues to demonstrate stable disease.

## 3 Discussion

AGCT is characterized by its indolent nature with one case report describing nearly three decades of treatment for recurrent disease (Teoh et al., 2010). While bleomycin/etoposide/cisplatin (BEP) or carboplatin/paclitaxel are clear first-line therapies, treatment options after progression from first-line therapy are less decisive, but often include chemotherapy or anti-hormonal therapies (Brown et al., 2024).

Hormonal therapies such as aromatase inhibitors and gonadotropin-releasing hormone (GnRH) agonists have shown objective response rates (ORR) between 10 and 20% and stable disease rates between 50 and 60%, making stable disease the predominant mode of measuring clinical benefit (Banerjee et al., 2021; Foster et al., 2023; Rothschild and Hillman, 2026). Other hormonal approaches that have demonstrated variable responses include combinations of tamoxifen and megestrol acetate, tamoxifen and leuprolide, and CDK 4/6 inhibitors with antiestrogen therapy (Keskin et al., 2012; Moon and Dorigo, 2021; Rothschild and Hillman, 2026).

Studies on chemotherapy for AGCT have also demonstrated variable ORR and often are limited by small sample sizes. A literature review evaluating 56 different chemotherapy regimens found an ORR of 30% and a stable disease rate of 58% (Brink et al., 2022). This review identified six studies evaluating platinum-based, taxane-based, and platinum-taxane combination regimens, with key studies including a retrospective analysis of BEP (ORR 22%) and a phase II trial of single-agent paclitaxel (ORR 29%) (van Meurs et al., 2014; Burton et al., 2016).

Although taxane-based therapy has shown clinical benefit in recurrent AGCT, published experience with docetaxel is extremely limited, and our case contributes to the existing literature demonstrating it as a potentially viable option. Preclinical data show that taxanes can inhibit steroidogenesis in granulosa cells by disorganizing microtubule bundles, with docetaxel having increased potency in promoting tubular polymerization and demonstrating increased cytotoxic activity compared to paclitaxel (Ringel and Horwitz, 1991; Chen et al., 1994). Despite our patient's prior progression to nab-paclitaxel, her subsequent response to docetaxel supports the concept of incomplete cross-resistance between taxanes and the potential benefit of taxane rechallenge with an alternative agent.

In a case report of recurrent epithelial ovarian cancer, weekly docetaxel demonstrated efficacy even after progression on paclitaxel, further demonstrating potential incomplete taxane cross-resistance (Komiyama et al., 2003). Another AGCT-specific case report describes a patient who initially achieved a complete response to paclitaxel but developed significant neurotoxicity. Following disease progression, the patient was transitioned to docetaxel and subsequently achieved a progression-free interval of 24 months (Shavit et al., 2012). Our patient similarly achieved a prolonged progression-free interval of approximately 36 months with a significant partial response that ultimately enabled tertiary cytoreductive surgery. Importantly, treatment remained well tolerated over 36 cycles, with minimal cumulative toxicity and without worsening of her baseline chemotherapy-induced peripheral neuropathy.

This case underscores several clinically relevant points. First, prolonged disease control with cytotoxic chemotherapy may be achievable in select patients with AGCT, even after multiple prior lines of therapy. Second, taxane rechallenge with an alternative agent may confer benefit in patients with prior taxane exposure or intolerance. Finally, durable systemic disease response can facilitate repeat cytoreductive surgery, which remains a cornerstone of management in recurrent AGCT and may contribute to prolonged disease control (Mangili et al., 2013).

Given the rarity of AGCT, prospective trials evaluating therapeutic sequencing and novel agents are needed. In the interim, carefully selected use of agents such as docetaxel may represent a reasonable option for patients with heavily pretreated recurrent disease.

## 4 Patient perspective

Informed consent was obtained from this patient for publication. At her most recent visit, the patient described her cancer journey as emotionally difficult and recognized the importance of maintaining a positive outlook throughout treatment. Despite fear surrounding recurrent disease, years of systemic therapy, and several major surgeries, she found that her children remained her primary motivation to continue treatment and “keep living.” She expressed gratitude for the time she was able to gain with her many different treatment regimens and reflected that focusing on family and hope helped her cope with the uncertainty of recurrent cancer.

## CRediT authorship contribution statement

**Maryam Ali:** Writing – review & editing, Writing – original draft, Investigation, Formal analysis, Data curation, Conceptualization. **Katherine Jane C. Chua:** Writing – review & editing, Resources, Data curation, Conceptualization. **Hui Chen:** Writing – review & editing, Supervision, Investigation, Formal analysis, Conceptualization.

## Declaration of competing interest

The authors declare that they have no known competing financial interests or personal relationships that could have appeared to influence the work reported in this paper.
